# Association of basal metabolic rate with mortality among cancer survivors: a cohort study emphasizing heterogeneity by gender and age

**DOI:** 10.1080/07853890.2026.2707731

**Published:** 2026-08-05

**Authors:** Guohui Zhang, Cunle Zhu, Xiaoyan Guo, Zhijian Xu, Xiangwei Wang

**Affiliations:** ^a^ Department of Urology, Affiliated Hospital of Guangdong Medical University, Zhanjiang, Guangdong Province, China; ^b^ Faculty of Chinese Medicine, Macau University of Science and Technology, Macau, China; ^c^ Department of Development and Planning, Affiliated Hospital of Guangdong Medical University, Zhanjiang, Guangdong Province, China

**Keywords:** Basal metabolic rate, cancer, survival, gender differences, age differences

## Abstract

**Background:**

The role of basal metabolic rate (BMR) on long-term survival outcomes in cancer survivors remains inconclusive. This study prospectively investigates the dose-response association between equation‑estimated BMR and mortality using data from a nationally representative cohort.

**Methods:**

Data from the United States (U.S.) National Health and Nutrition Examination Survey (NHANES, 2001–2018) were analyzed, and BMR was compared between cancer survivors (*n* = 3,434) and cancer-free adults (*n* = 42,425). BMR’s nonlinear association with all-cause and cause-specific mortality among survivors was assessed using multivariable Cox regression and restricted cubic spline, stratified by age and gender.

**Results:**

The BMR of cancer survivors was significantly lower than that of participants without cancer. A total of 1,145 deaths occurred during a median follow-up of 79 months. In the overall cohort of survivors, a higher BMR was associated with reduced prevalence of all-cause and cardiac mortality, but not cancer-specific mortality. Stratified analysis showed significant associations only in those aged ≥60 years. Among older females, a U-shaped relationship was identified for all-cause mortality with an inflection point at 1546.8 kcal/d; below this threshold, higher BMR was associated with reduced mortality, while above it, risk increased. In contrast, a linear protective association was observed in older males. No significant associations were found for survivors aged <60 years.

**Conclusions:**

Equation-estimated BMR is lower in cancer survivors and shows age- and sex-specific associations with long-term mortality, particularly in older survivors. These findings suggest that BMR may reflect underlying metabolic reserve and may provide insight into subgroup heterogeneity in survivorship, although these findings are exploratory and further validation is needed before any clinical application.

## Background

The population of cancer survivors worldwide is rapidly growing [1]. Approximately 18.6 million individuals in the U.S. are living with a history of cancer, with 2.04 million new cases anticipated annually [2,3]. Driven by continued advances in early detection and treatment, the population of cancer survivors is projected to reach over 22 million by 2035 [3]. Moreover, cancers and cancer treatments cause severe physical disabilities and increased symptom burdens for survivors [4], ultimately leading to a shortened life expectancy [5]. Therefore, it is imperative to identify modifiable strategies that improve the long-term health of cancer survivors.

The basal metabolic rate (BMR), which represents the minimal resting energy required for vital physiological functions and accounts for approximately 60–70% of daily energy consumption, is a fundamental component of bodily energy homeostasis and metabolic health [6]. It decreases with age or weight loss, and increases with muscle mass [7]. Prior research has implicated BMR in a range of critical health outcomes, including cancer occurrence and development [8,9], ageing [10], longevity [11], cardio-metabolic health [12], insulin resistance, diabetes [13] and functional disability [14]. However, evidence regarding the impact of BMR on long-term survival outcomes remains incompletely understood. Epidemiological studies on the association between BMR and all-cause mortality in the general population have yielded conflicting results [15–18]. More importantly, among the growing population of cancer survivors, the evidence is particularly limited and inconsistent. On the one hand, findings from several cohort studies consistently indicate that a higher BMR is a protective factor linked to improved overall survival for specific cancer types [19–21]. In contrast, separate prospective studies implicate elevated BMR in the development of cachexia and subsequent shorter survival [22,23]. This paradox underscores the unresolved controversy regarding the overall effect of BMR in cancer patients.

Despite growing interest in BMR and health outcomes, evidence regarding its prognostic relevance among cancer survivors remains limited and inconsistent and prior studies have largely been restricted to specific cancer types, small clinical cohorts, or non-representative samples, with limited evaluation of potential nonlinear associations and effect modification by age and gender. Therefore, using a nationally representative cohort from NHANES linked to mortality follow-up, we aimed to (1) compare BMR between cancer survivors and cancer-free adults to characterize metabolic alterations associated with cancer history and (2) prospectively examine the association of BMR with mortality among cancer survivors, where BMR may serve as a potential prognostic factor. We hypothesized that BMR would be associated with long-term mortality risk among cancer survivors, particularly for all-cause and cardiovascular mortality and that this association might be modified by age and sex and could exhibit a nonlinear pattern. By integrating nationally representative data, cause-specific mortality and formal assessment of nonlinearity and effect modification, this study provides additional nationally representative evidence on the prognostic relevance of BMR in cancer survivorship.

## Methods

### Study design and population

This study included participants from a prospective cohort in the NHANES to investigate the association between BMR and mortality among cancer survivors. NHANES is a nationally representative survey administered by the National Center for Health Statistics (NCHS), part of the Centers for Disease Control and Prevention (CDC). It is designed to evaluate the health and nutritional status of the noninstitutionalized U.S. population. A comprehensive description of the study design and methodology has been provided previously(www.cdc.gov/nchs/nhanes/). The survey protocol was approved by the NCHS Research Ethics Review Board. Written informed consent was obtained from all participants in the NHANES study.

This study analyzed nine cycles (2001–2018) of NHANES data, with a total of 91,351 participants surveyed. After excluding participants aged <18 years (*n* = 37,595), those with missing BMR data (*n* = 3,453), missing cancer diagnosis information (*n* = 3,392), or incomplete follow-up records (*n* = 102), the final analytical dataset for this study was 45,859 adults, included 3,434 cancer survivors and 42,425 cancer-free individuals (Figure S1). Cancer-free individuals were included for cross-sectional BMR comparison only; they were excluded from survival analyses as they lack a cancer diagnosis date to define follow-up onset.

### Cancer diagnosis

Data on cancer diagnosis, including cancer type and age at first diagnosis, were collected through a validated questionnaire. The participants were asked, ‘Have you ever been told that you have cancer or malignant tumors?’. Those who answered ‘yes’ were defined as cancer survivors. They were further asked, ‘What type of cancer was it?’ and ‘How old were you when you were first diagnosed with this cancer?’. Years since the initial cancer diagnosis were calculated as the difference between age at interview (baseline) and age at first diagnosis. The analysis accounted for up to three independent cancer diagnoses per participant. Participants with only nonmelanoma cancer or unknown-type skin cancers were excluded from the analyses.

### Mortality ascertainment

The death information of all participants was obtained through linkage to the National Death Index through 31 December 2019. The International Statistical Classification of Diseases, Tenth Revision (ICD-10) was adopted to encode the cause of death. The main mortality outcomes considered in this study included all-cause mortality, cardiovascular mortality (codes I00-I09, I11, I13 and I20-I51), cancer-specific mortality (codes C00-C97) and mortality from other causes. The follow-up period was calculated from the date of the NHANES interview to the date of death or December 31, 2019, whichever came first. For participants who were alive at the end of follow-up, survival time was censored on December 31, 2019.

### BMR assessment

In the NHANES, BMR was estimated using the Mifflin-St Jeor equation on the basis of age at interview, gender, weight and height. The detailed calculation was as follows. For men, BMR (kcal/day) = 9.99 × weight (kg) + 6.25 × height (cm) − 4.92 × age (years) + 5. For women, BMR (kcal/day) = 9.99 × weight (kg) + 6.25 × height (cm) − 4.92 × age (years) − 161.

### Covariates

A series of covariates potentially associated with BMR and cancer survivor mortality were selected to minimize bias in the analysis. The specific covariates and their classifications are as follows:

Demographic covariates included age at interview(<60 years or ≥60 years), gender (male/female), race/ethnicity (Mexican American, Non-Hispanic White, Non-Hispanic Black, Other Hispanic or Other Race), educational attainment (less than high school, high school graduate or more than high school), marital status (married/cohabiting, widowed/divorced/separated or never married) and income level based on the poverty-income ratio (PIR: low [<1], moderate [1.3–3.5] or high [>3.5]).

Lifestyle variables included smoking status (non-smoker, current smoker, or former smoker), alcohol consumption (non-drinker or drinker) and physical activity (active or inactive). Specifically, a nonsmoker was defined as an individual who had smoked fewer than 100 cigarettes in their lifetime, while a former smoker was defined as one who had quit after smoking more than 100 cigarettes. For alcohol consumption, a non-drinker was defined as consuming ≤12 drinks annually and a drinker was defined as consuming >12 drinks annually. The physical activity status was determined based on participation in moderate-to-vigorous leisure-time physical activities, assessed over the previous 30 days in the 2001–2006 cycles or over a typical week in the 2007–2018 cycles of NHANES.

Health conditions included hypertension, diabetes, cardiovascular disease (CVD), body mass index (BMI) and waist circumference. Hypertension was defined as having a prior clinical diagnosis, current use of antihypertensive medication, or an average blood pressure ≥140/90 mmHg. Diabetes mellitus was defined by a self-reported physician diagnosis, use of antidiabetic medication, a fasting plasma glucose level ≥7.0 mmol/L, or a glycated haemoglobin (HbA1c) level ≥6.5%. CVD was defined based on self-reported physician diagnoses of congestive heart failure, coronary heart disease, angina, myocardial infarction, or stroke, which is consistent with definitions used in prior cohort studies. BMI was categorized as <25 kg/m^2^, 25–30 kg/m^2^ or ≥30 kg/m^2^.

Cancer-specific covariates were also included to account for cancer history and severity. These comprised the time since the first cancer diagnosis (categorized as <5 years, 5–10 years or ≥10 years), the age at the first cancer diagnosis (categorized as <39 years, 40–60 years or ≥60 years) and the number of distinct cancer types diagnosed (categorized as 1 or ≥2).

### Statistical analysis

All analyses were conducted in accordance with the NHANES analytical guidelines to generate nationally representative estimates. Multiple imputation has been implemented to address the issue of missing covariate data, with the degree of missing data detailed in Supplementary Table 1. To facilitate a more meaningful interpretation, BMR was scaled to represent a 50-kcal/day increment in the analysis of its association with survival. The sample size (weighted percentage) and median BMR (interquartile range [IQR]) were estimated based on participants’ sociodemographic and lifestyle factors, health status, as well as cancer type and history (limited to cancer survivors only). Four multivariate Cox proportional hazards regression models were established among cancer survivors to estimate the hazard ratios (HRs) and 95% confidence intervals of the association between BMR and all-cause mortality and cause specific mortality: Model 1: adjusted for no covariates; Model 2: adjusted for gender, age at interview, race, marital status, educational attainment and PIR; Model 3: additionally adjusted for waist circumference, BMI, physical activity, drinking status and smoking status; Model 4: additionally adjusted for hypertension, diabetes, CVD, years since first diagnosis of cancer, age of first cancer diagnosis and number of cancer types.

To examine the potential nonlinear relationship between BMR and mortality among cancer survivors, we first employed a generalized additive model (GAM) with smooth curve fitting within the Cox proportional hazards framework. If a nonlinear relationship was suggested, an inflection point was determined using a recursive algorithm and the association was further characterized using a two-piecewise Cox model to calculate the hazard ratios on both sides of the inflection point.

All analyses were conducted using SPSS (statistical product and service solutions) software. The statistical test adopts a two-tailed test, and a p-value less than 0.05 is considered statistically significant. Unless otherwise specified, age refers to age at interview (baseline), whereas age at diagnosis refers to age at first cancer diagnosis.

## Results

### Baseline characteristics of study population

The baseline characteristics of participants categorized by cancer status are presented in Table 1. A total of 42,425 adults without cancer (representing 194,355,196 US adults without cancer) and 3,434 cancer survivors (representing 15,081,261 US cancer survivors) were included in the analysis. Compared with participants without cancer, cancer survivors were more likely to be older, female, non-Hispanic White, have higher educational attainment and household income, and a history of smoking. They also had a higher prevalence of hypertension, diabetes and cardiovascular disease, but engaged in less physical activity(all *p* < 0.001). Among cancer survivors, the median (interquartile range, IQR) age at first cancer diagnosis was 58 (42,68) years, the median (IQR) age at interview was 68 (57,78) years and the median time from first diagnosis to interview was 8 (3–15) years (Table 1).

**Table 1.** Sample size and BMR according to characteristics among U.S. adults without cancer and cancer survivors.

	No. of participants (weighted %)			BMR(kcal/d), mean (IQR)			
Characteristic	Adults without cancer	Cancer survivor	*p*-Value	Adults without cancer	*p*-Value	Cancer survivor	*p*-Value
All	42425	3434	–	1526.1 (1313.5, 1739.2)		1418.8 (1224.9, 1610.9)	< 0.001
Weighted NO.	194355196	15081261	–	194355196	–	15081261	–
Age at interview, y, Mean (IQR)	46 (33,62)	68 (57,78)	< 0.001	–	–	–	–
Age at interview			< 0.001	–	< 0.001	–	<0.001
<60years	29982 (79.2)	986 (38.5)		1589.2 (1373.8, 1789.3)		1478.5 (1307.6, 1674.3)	
≥60 years	12443 (20.8)	2448 (61.5)		1378.0 (1178.8, 1582.9)		1396.2 (1187.3, 1582.3)	
Gender			< 0.001		< 0.001		<0.001
Male	20509 (48.7)	1521 (38.1)		1704.7 (1558.7, 1863.7)		1576.8 (1432.7, 1740.1)	
Female	21916 (51.3)	1913 (61.9)		1338.2 (1200.9, 1501.6)		1266.3 (1120.7, 1440.6)	
Race			< 0.001		< 0.001		<0.001
Mexican American	7496 (8.9)	275 (3.1)		1491.7 (1297.7, 1679.0)		1359.2 (1195.0, 1529.3)	
Other Hispanic	3700 (5.7)	213 (3.0)		1462.8 (1267.0, 1669.4)		1304.9 (1179.9, 1489.4)	
Non-Hispanic White	17481 (65.8)	2135 (82.1)		1547.2 (1323.0, 1769.2)		1417.3 (1219.9, 1602.0)	
Non-Hispanic Black	9337 (11.9)	618 (7.5)		1593.7 (1392.7, 1797.0)		1509.4 (1343.1, 1689.1)	
Other Race	4411 (7.6)	193 (4.3)		1413.6 (1208.9, 1633.0)		1339.3 (1149.8, 1540.2)	
Educational attainment			0.026		< 0.001		<0.001
<High school	11208 (17.1)	842 (15.3)		1474.9 (1271.2, 1666.6)		1388.5 (1204.8, 1578.8)	
High school	9848 (24.1)	800 (23.1)		1552.4 (1325.1, 1760.3)		1410.4 (1223.2, 1595.4)	
>High school	21369 (58.9)	1792 (61.6)		1545.6 (1332.4, 1762.5)		1438.7 (1241.5, 1633.8)	
Marital status			< 0.001		< 0.001		<0.001
Married/living with partner	25507 (63.4)	2011 (63.9)		1539.9 (1330.1, 1746.3)		1456.3 (1282.7, 1647.9)	
Widowed/divorced/separated	8875 (17.3)	1188 (29.8)		1398.0 (1201.1, 1630.5)		1333.3 (1132.9, 1519.5)	
Never married	8043 (19.3)	235 (6.4)		1617.4 (1402.6, 1806.4)		1461.2 (1289.8, 1661.9)	
PIR			< 0.001		< 0.001		<0.001
Low(<1)	9166 (15.1)	580 (11.7)		1506.1 (1304.3, 1704.8)		1399.3 (1217.4, 1561.6)	
Medium(1–3)	17942 (36.9)	1520 (36.5)		1517.0 (1306.4, 1730.2)		1407.0 (1210.4, 1583.4)	
High(>3)	15317 (48.0)	1334 (51.8)		1551.4 (1326.7, 1768.0)		1444.6 (1245.6, 1653.1)	
Drinking status			< 0.001		< 0.001		<0.001
No	12230 (23.8)	1138 (28.7)		1413.2 (1224.0, 1643.3)		1349.4 (1147.6, 1540.8)	
Yes	30195 (76.2)	2296 (71.3)		1569.0 (1356.5, 1768.9)		1449.5 (1265.3, 1637.3)	
Smoking status			< 0.001		< 0.001		<0.001
Never smoker	23624 (55.1)	1519 (44.2)		1481.6 (1278.4, 1718.8)		1365.2 (1175.0, 1563.7)	
Former smoker	9663 (23.1)	1345 (38.1)		1554.5 (1352.3, 1749.2)		1466.2 (1289.0, 1650.5)	
Current smoker	9138 (21.8)	570 (17.7)		1588.8 (1379.5, 1768.5)		1419.5 (1257.7, 1606.9)	
Hypertension			< 0.001		< 0.001		0.499
No	25603 (65.4)	1231 (41.7)		1535.3 (1332.1, 1735.9)		1407.2 (1236.5, 1597.4)	
Yes	16822 (34.6)	2203 (58.4)		1510.6 (1280.5, 1745.1)		1426.7 (1222.2, 1620.8)	
Diabetes			< 0.001		< 0.001		< 0.001
No	27860(89.0)	2618 (80.2)		1522.7 (1310.8, 1735.2)		1399.2 (1209.9, 1591.8)	
Yes	7696 (11.0)	816 (19.8)		1540.3 (1326.4, 1762.7)		1475.8 (1287.8, 1673.0)	
CVD			< 0.001		< 0.001		0.912
No	38395 (92.7)	2578 (79.5)		1531.7 (1317.7, 1743.6)		1417.6 (1223.4, 1612.4)	
Yes	4030 (7.3)	856 (20.4)		1479.1 (1271.8, 1688.7)		1421.5 (1233.4, 1606.6)	
Physical activity			< 0.001		< 0.001		< 0.001
Inactive	20768 (42.2)	1955 (50.2)		1496.5 (1289.0, 1710.4)		1401.3 (1202.6, 1599.1)	
Active	21657 (57.8)	1479 (49.8)		1555.9 (1338.6, 1760.2)		1440.3 (1254.3, 1624.7)	
WC	98.7 ± 16.1	101.4 ± 15.4	< 0.001	–	–	–	–
BMI(kg/m^2^), Mean ± SD	29.0 ± 6.9	28.9 ± 6.5	0.895	–	–	–	–
BMI			0.072		< 0.001		< 0.001
<25	12593 (31.0)	964 (28.6)		1339.3 (1187.6, 1545.2)		1243.5 (1069.7, 1404.2)	
25–30	14201 (33.1)	1205 (33.8)		1510.2 (1321.1, 1702.7)		1407.1 (1224.2, 1560.3)	
≥30	15631 (35.9)	1265 (37.7)		1695.2 (1485.9, 1921.5)		1591.0 (1403.3, 1779.1)	
Cancer type							< 0.001
Prostate	–	602 (11.6)	–	–	–	1560.8 (1429.6, 1716.3)	
Breast	–	586 (18.5)	–	–	–	1222.9 (1091.6, 1386.9)	
Colorectal	–	240 (5.5)	–	–	–	1401.3 (1187.0, 1631.0)	
Gynecologic	–	507 (16.6)	–	–	–	1359.1 (1196.7, 1511.1)	
Melanoma	–	210 (8.6)	–	–	–	1481.5 (1296.7, 1643.6)	
Lung		89 (2.34)				1404.8 (1227.5, 1594.2)	
Bladder		74 (1.87)				1501.6 (1372.4, 1662.2)	
Other	–	662 (20.9)	–	–	–	1454.9 (1269.7, 1670.8)	
multiple cancers	–	464 (14.1)	–	–	–	1398.5 (1204.0, 1602.6)	
No. of cancer types	–		–	–	–		0.236
1	–	2970 (85.9)	–	–	–	1421.4 (1228.9, 1611.9)	
≥2	–	464 (14.1)	–	–	–	1398.5 (1204.0, 1602.6)	
Time since cancer diagnosis, y (Median (IQR))	–	8 (3,15)	–	–	–		< 0.001
<5	–	1171 (32.3)	–	–	–	1431.0 (1250.2, 1626.7)	
5–10	–	780 (22.6)	–	–	–	1448.7 (1257.0, 1634.5)	
≥10	–	1483 (45.1)	–	–	–	1391.5 (1194.7, 1584.9)	
Age at cancer diagnosis, y (Median (IQR))	–	58 (42,68)	–	–	–		< 0.001
0–39	–	735 (27.2)	–	–	–	1450.9 (1279.4, 1639.2)	
40–60	–	1150 (37.1)	–	–	–	1424.5 (1226.8, 1634.3)	
≥60	–	1549 (35.7)	–	–	–	1401.2 (1192.1, 1579.2)	

Weighted to be nationally representative. BMR = basal metabolic rate; IQR = interquartile range; PIR = poverty income ratio, BMI = body mass index; CVD = cardiovascular disease; WC = waist circumference; SD = standard deviation.

### Distribution of BMR in adults without cancer and cancer survivors

**Figure 1.**
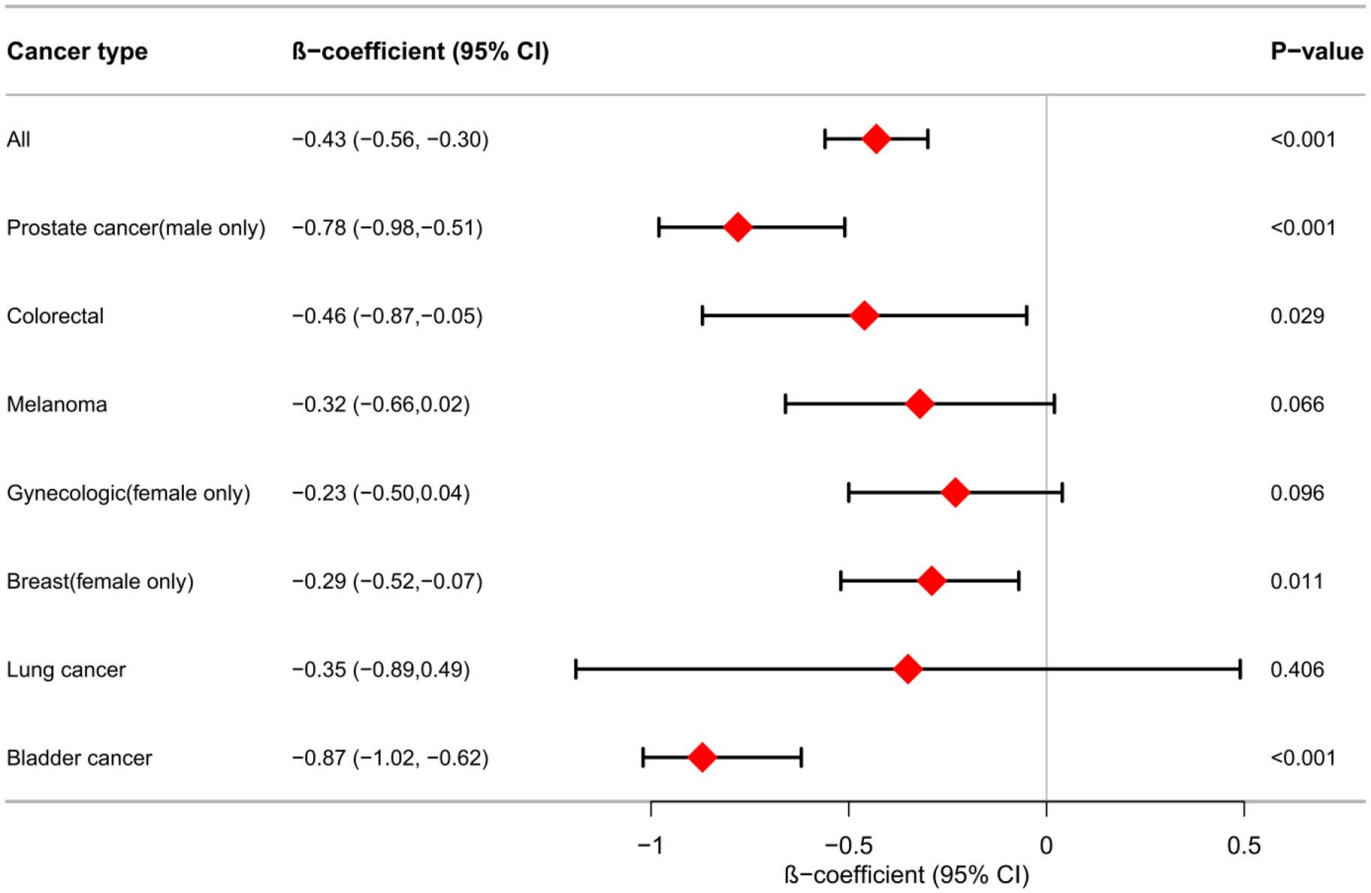
Stratified β-coefficients and 95% confidence intervals for BMR for cancer survivors vs individuals without cancer. β-coefficients were estimated for a 50-unit increase in BMR. Adjusted for gender, age at interview, race, marital status, educational attainment, poverty income ratio(PIR), waist circumference, body mass index (BMI), physical activity, drinking status, smoking status, hypertension, diabetes and cardiovascular disease (CVD).

BMR level of cancer survivors was significantly lower than that of participants without cancer(1418.8 vs 1526.1 kcal/day; Table 1). After adjusting for sociodemographic and lifestyle status as well as health conditions, the BMR of cancer survivors was significantly lower than that of adults without cancer (β=-0.43, 95% CI=-0.56 to −0.30, *p* < 0.001) (Figure 1). This difference was particularly evident among survivors of bladder, prostate, breast and colorectal cancer (Figure 1). Within survivors, BMR differed by key demographic and anthropometric characteristics (Table 1), with lower values observed in female and in those aged ≥60 years at interview. Associations of comorbidities with BMR after multivariable adjustment are presented in supplementary Table S2.

### BMR and mortality in cancer survivors

During a median follow-up of 79 (IQR: 40,129) months from the NHANES interview, a total of 1,145 death events were recorded, including 407 (35.5%) cancer deaths and 230 (20.1%) cardiac mortalities. In fully adjusted Cox models, higher BMR was associated with a lower hazard of all-cause mortality (per 50 kcal/day increase: HR 0.92, 95% CI 0.89–0.96) and cardiovascular mortality (HR 0.87, 95% CI 0.81–0.93), whereas no significant association was observed with cancer-specific mortality (Table 2). Consistent results were observed when BMR was analyzed in categories, with lower hazards across higher BMR groups for all-cause and cardiovascular mortality (Table 2).

**Table 2.** Multivariable cox proportional hazards analysis of the association of BMR with all-cause and cause-specific mortality(weighted).

Mortality outcome	Death, n/N	Weighted death, No. (%)	Model 1	Model 2	Model 3	Model 4
			HR(95% CI)	HR(95% CI)	HR(95% CI)	HR(95% CI)
**All cause**						
Per 50 kcal/d increase	1145/3434	2779643(25.1)	0.96(0.94,0.97)***	0.93(0.91,0.95)***	0.90(0.87,0.93)***	0.92(0.89,0.96)***
<1225.1 kcal/d	347/859	1254952 (34.4)	Reference	Reference	Reference	Reference
1225.1–1418.8 kcal/d	264/858	793374 (20.8)	0.56(0.45,0.69)***	0.55(0.44,0.69)***	0.53(0.42,0.68)***	0.61(0.48,0.77)***
1418.8–1611.0 kcal/d	286/858	823454 (23.3)	0.61(0.49,0.76)***	0.47(0.37,0.61)***	0.46(0.35,0.62)***	0.57(0.42,0.76)***
≥1611.0 kcal/d	248/859	907863 (22.2)	0.60(0.50,0.73)***	0.44(0.33,0.58)***	0.42(0.28,0.62)***	0.58(0.39,0.87)**
p for trend	N/A	N/A	<0.001	<0.001	<0.001	0.02
Cardiac disease						
Per 50 kcal/d increase	230/3434	730906(4.8)	0.94(0.91,0.98)**	0.93(0.88,0.98)**	0.83(0.77,0.89)***	0.87(0.81,0.93)***
<1225.1 kcal/d	68/859	244141 (6.7)	Reference	Reference	Reference	Reference
1225.1–1418.8 kcal/d	53/858	166089 (4.4)	0.61(0.44,0.92)*	0.60(0.35,1.03)	0.43(0.24,0.76)**	0.48(0.27,0.85)*
1418.8–1611.0 kcal/d	62/858	177283 (5.0)	0.68(0.45,1.02)	0.54(0.30,0.95)*	0.31(0.16,0.62)***	0.39(0.19,0.81)*
≥1611.0 kcal/d	47/859	143393 (3.5)	0.49(0.32,0.75)***	0.40(0.21,0.77)**	0.16(0.07,0.39)***	0.24(0.09,0.61)**
p for trend	N/A	N/A	0.003	0.007	<0.001	0.007
Cancer						
Per 50 kcal/d increase	407/3434	1393446(9.2)	1.00(0.97,1.02)	0.96(0.92,0.99)*	0.95(0.90,1.01)	0.99(0.93,1.05)
<1225.1 kcal/d	101/859	356910 (9.8)	Reference	Reference	Reference	Reference
1225.1–1418.8 kcal/d	100/858	309643(8.1)	0.78(0.56,1.09)	0.69(0.49,0.98)*	0.75(0.51,1.10)	0.87(0.60,1.27)
1418.8–1611.0 kcal/d	97/858	289487 (8.2)	0.77(0.54,1.11)	0.51(0.33,0.80)*	0.60(0.36,1.00)	0.76(0.44,1.32)
≥1611.0 kcal/d	109/859	437407 (10.7)	1.03(0.75,1.43)	0.59(0.38,0.92)*	0.75(0.40,1.41)	1.12(0.58,2.14)
p for trend	N/A	N/A	0.776	0.032	0.399	0.752
All other causes						
Per 50 kcal/d increase	508/3434	1655290 (11.0)	0.93(0.90,0.95)***	0.91(0.88,0.94)***	0.88(0.84,0.92)***	0.90(0.86,0.94)***
<1225.1 kcal/d	178/859	653901 (17.9)	Reference	Reference	Reference	Reference
1225.1–1418.8 kcal/d	111/858	317642 (8.3)	0.42(0.31,0.58)***	0.44(0.31,0.63)***	0.44(0.30,0.64)***	0.49(0.34,0.70)***
1418.8–1611.0 kcal/d	127/858	356684 (10.1)	0.50(0.39,0.65)***	0.43(0.32,0.59)***	0.44(0.30,0.66)***	0.52(0.35,0.77)**
≥1611.0 kcal/d	92/859	327063 (8.0)	0.41(0.29,0.59)***	0.35(0.22,0.57)***	0.36(0.20,0.64)***	0.45(0.25,0.82)**
p for trend	N/A	N/A	<0.001	<0.001	0.002	0.023

Model 1: adjusted for no covariates.

Model 2: adjusted for gender, age at interview, race, marital status, educational attainment and poverty income ratio (PIR).

Model 3: additionally adjusted for waist circumference, body mass index (BMI), physical activity, drinking status and smoking status.

Model 4: additionally adjusted for hypertension, diabetes, ardiovascular disease (CVD), years since first diagnosis of cancer, age of first cancer diagnosis and number of cancer types.

BMR = basal metabolic rate; HR = hazard ratio; CI = confidence interval; N/A = not applicable.

Significance levels: ***p < 0.001, **p < 0.01, *p < 0.05.

Associations between BMR and all-cause mortality were generally consistent across most demographic, lifestyle and comorbidity subgroups (Figure S3), with no significant interactions observed for these factors. In contrast, the association was significantly modified by age and gender (p for interaction <0.05; Figure S3). Subsequent subgroup analyses confirmed these effect modifications and revealed distinct patterns (Table S3). A strong protective association between higher BMR and all-cause mortality was confirmed in females aged ≥60 years (HR =0.85; *p* < 0.001). A consistent, although borderline significant, inverse association was observed in older males (HR = 0.95; *p* = 0.055), which was further substantiated by a significant dose-response relationship across BMR quartiles (p for trend =0.042). In contrast, no such association was observed in either gender among younger adults (<60 years). Similar gender- and age-specific patterns were observed for cardiovascular mortality, while cancer-specific mortality remained null across subgroups (Table S3). Additional cancer-type-specific estimates and interaction tests are provided in supplementary Figure S2, and interactions by time since diagnosis are reported therein.

### Detection of nonlinear relationships

**Figure 2.**
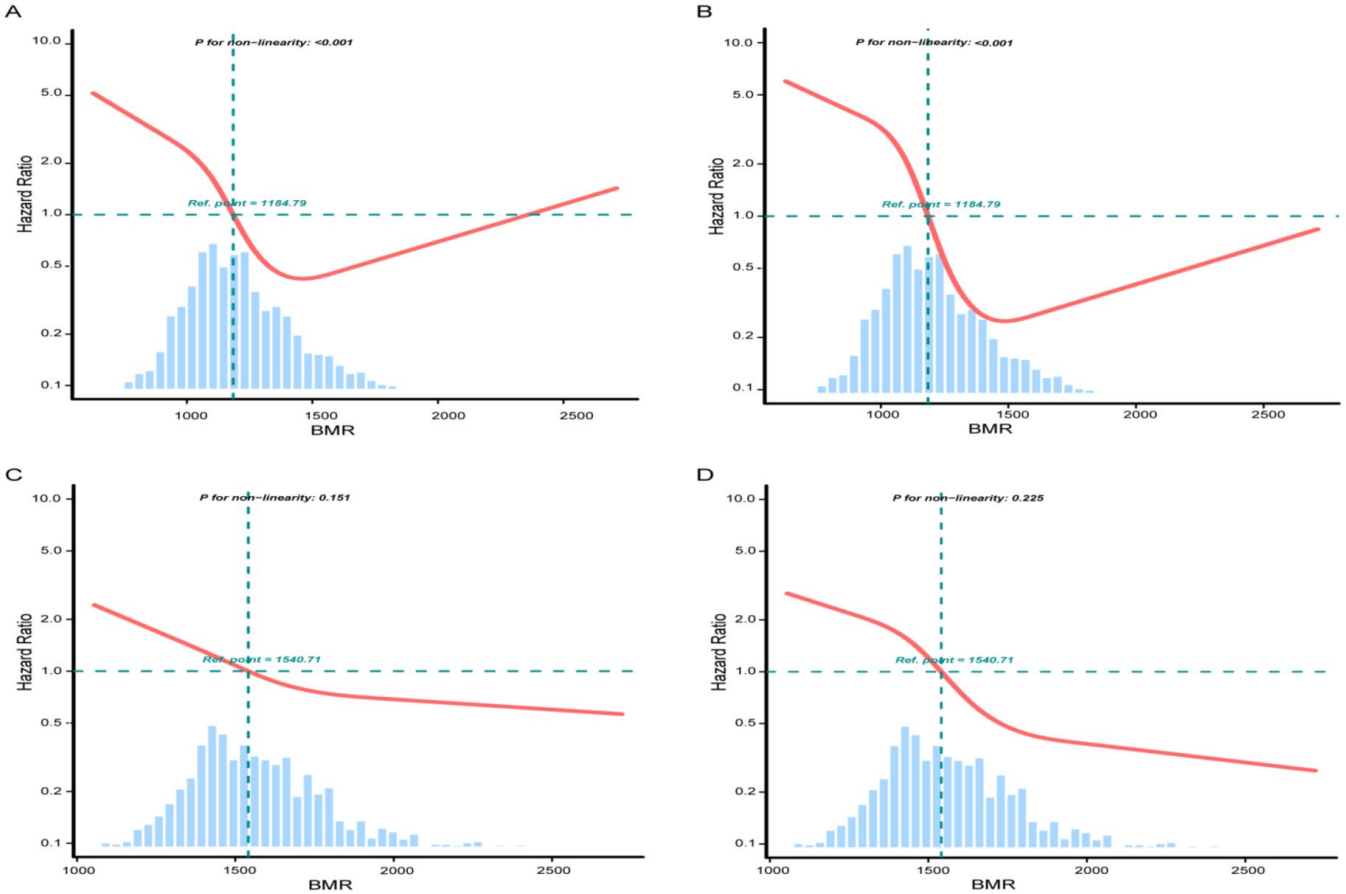
Dose-response association of basal metabolic rate (BMR) with all-cause and cardiovascular mortality among US cancer survivors, stratified by gender and age at interview (≥60 years). (A) All-cause mortality in females. (B) Cardiovascular mortality in females. (C) All-cause mortality in males. (D) Cardiovascular mortality in males.

Restricted cubic spline analyses indicated a significant nonlinear association between BMR and both all-cause and cardiovascular mortality among females aged ≥60 years at interview (both p for nonlinearity <0.001; Figure 2), whereas no significant departure from linearity was observed among males aged ≥60 years (Figure 2). In older females, a U-shaped association was identified for all-cause mortality with an inflection point at 1546.8 kcal/day: below this threshold, each 50 kcal/day increase in BMR was associated with a lower hazard of all-cause mortality (HR = 0.84), whereas above it the hazard increased (HR = 1.49) (Table S4). For cardiovascular mortality in older females, results suggested a similar threshold pattern, although estimates above the inflection point were imprecise due to a small number of events (Table S4).

## Discussion

In this U.S. nationally representative cohort study, BMR levels were significantly lower in cancer survivors, particularly survivors of bladder, prostate, colorectal and breast cancer, than in adults without cancer. Over a median follow-up of 79 months, elevated BMR was associated with decreased all-cause and cardiac mortality among cancer survivors, whereas no significant association was observed with cancer-specific mortality. These two findings address fundamentally different questions: the former compares cancer survivors with the general population, while the latter evaluates the prognostic value of BMR within the survivor population itself.

However, the prognostic value of BMR was not uniform but was profoundly modified by age and gender. To our knowledge, this is the first study to report a U-shaped relationship between BMR and all-cause mortality in female cancer survivors aged ≥60 years, with an inflection point at 1546.8 kcal/d: risk decreased below this threshold but increased above it. A similar non-linear association pattern was also observed for cardiac mortality in this subgroup. In contrast, the association in males aged ≥60 years appeared modest and approximately linear, and no significant associations were observed in survivors aged <60 years.

The median BMR of U.S. cancer survivors was 1418.8 kcal/d, significantly lower than that of non-cancer participants and this difference remained statistically significant after comprehensive adjustment for covariates. This finding is consistent with observations from previous studies [24]. The BMR, as an indicator of energy metabolism, is influenced by multiple factors, including body composition, cancer type, treatment methods, age, gender and hormone levels, making it a complex situation [25,26]. Given that BMR is primarily determined by fat-free mass [27], this systemic depression in energy expenditure likely indicates a higher prevalence of sarcopenia or relative muscle loss in the cancer survivor population [28] - a condition strongly linked to functional decline, poorer treatment tolerance and reduced survival [28,29]. This metabolic alteration transcends specific cancer types, pointing to a common adverse sequelae of the cancer experience that may critically impair physiological reserve [30].

This sexual dimorphism observed in our study may be attributed to distinct metabolic and hormonal profiles. Significant gender differences have been documented in various metabolic processes, including glycolysis and fatty acid, bile acid and xenobiotic metabolism [31]. Stable oestrogen levels are protective against survival in women [32]. Conversely, the hormonal transition during menopause adversely affects overall energy expenditure and basal metabolic rate [33], with implications for cancer survival outcomes [34]. The absence of an association in younger survivors may be explained by their more favourable body composition and greater metabolic reserve [35,36], which could buffer against variations in BMR. Additionally, the observed age difference could also be attributable to the lower number of mortality events in the younger cohort. These findings underscore the critical importance of considering gender and age when evaluating the protective role of BMR against mortality in cancer survivors, indicating that tailored strategies may be needed to optimize BMR in different populations.

Although the underlying mechanism of the complex association between BMR and mortality still needs to be investigated, our findings are biologically plausible based on the available evidence. In addition to genetic disorders, cancer is increasingly viewed as a metabolic disease [37]. A lower BMR leads to reduced energy expenditure, which in turn represents a state of metabolic imbalance [13]. Scientific management and maintenance of energy metabolism may therefore contribute to improved prognosis [38]. In genetics, BMR is associated with multiple phenotypes, including biomarkers, diseases and health-related outcomes. For certain phenotypes, there are gender differences that typically affect the strength of effects [39]. Evidence from animal and human population studies indicates that a low BMR contributes to metabolic impairment by promoting insulin resistance and diabetes [13,40], a process characterized by accelerated weight gain, hyperglycaemia and dyslipidemia [13]. Crucially, both insulin resistance and diabetes are established high-risk factors for all-cause and cardiac disease mortality in cancer patients [41,42]. Approximately half of patients with advanced cancer experience cachexia, a multifactorial syndrome characterized by inadequate food intake, progressive weight loss, depletion of muscle mass and energetic inefficiency of mitochondria, leading to metabolic derangements that ultimately decrease survival [43,44]. Other physiologic mechanisms might also be involved, and future studies are needed to explore how BMR influences health outcomes in cancer survivors.

Given its modifiable nature and complex association with survival outcomes, BMR represents an important research target for improving patient prognosis. A nested case–control study from southern China, involving 12,608 participants aged 35 years or older with an average follow-up period of 5.6 years, reported that a 1 standard deviation increase in BMR was associated with a 20% decrease in all-cause mortality among older males [15]. This study confirmed the protective role of BMR against all-cause mortality in the general population; however, its specific association with cancer survivors remains unexplored. Recent studies have explored the relationship between BMR and mortality in patients with cancer and survivors. In a retrospective cohort study of 521 Asian gastric cancer patients, BMR was identified as a significant independent predictor for overall survival, with higher BMR values demonstrating a linear correlation with reduced mortality [21]. Similar patterns were corroborated by other retrospective analyses conducted in distinct single-cancer cohorts, focusing on oesophageal and gastric cancers, respectively [19,20]. Collectively, these findings form a coherent body of evidence that aligns with the association identified in our present study. However, these studies were limited by their single-centre, cross-sectional design with a small sample size, which constrains the generalizability of the findings. Furthermore, existing research has primarily focused on specific cancer types and often lacks the perspective of long-term survivorship.

Although we observed marked age- and gender-specific heterogeneity, these findings should not be interpreted as recommending BMR as a direct therapeutic target. Because BMR is largely determined by body composition and other physiologic factors, it is not easily amenable to direct clinical manipulation. Instead, our results suggest that equation-estimated BMR may serve as an integrative marker of underlying metabolic reserve among older cancer survivors and may help identify subgroups who could benefit from closer evaluation of nutritional status and body composition and from evidence-based interventions targeting these modifiable conditions. Future studies are warranted to validate these associations and to clarify how BMR-related metabolic phenotypes can be incorporated into survivorship assessment frameworks.

All BMR values in this study were estimated using the Mifflin-St Jeor equation, which was developed in healthy populations. This equation has not been validated in cancer survivors, whose metabolic state may be substantially altered by the disease itself and by its treatments. Therefore, the estimated BMR may be systematically biased or imprecise at the individual level. Consequently, our findings should be interpreted as exploratory associations with a calculated metabolic index, not with a directly measured physiological parameter. They do not support the use of estimated BMR as a clinical biomarker or an independent prognostic tool. Direct measurement of resting energy expenditure in future studies is essential to determine whether the observed age- and sex-specific patterns reflect true biological relationships.

A key strength of this study is the use of a nationally representative sample of U.S. cancer survivors, which enhances the generalizability of our findings to the broader population. However, several limitations should be considered. First, owing to the observational study design and the absence of pre-diagnosis BMR measurements, we cannot determine whether the lower BMR observed in cancer survivors reflects a pre-existing metabolic characteristic or a consequence of the disease and its treatment. This limitation precludes any inference regarding the relationship between pre-disease BMR and the risk of cancer development. Future prospective studies with longitudinal BMR assessments before and after cancer diagnosis are warranted to establish causality. Second, despite extensive adjustment for potential confounders, residual confounding cannot be completely excluded, particularly because some relevant clinical and biological factors were not captured in NHANES. Although we were able to account for cancer type, age at diagnosis, time since diagnosis and multiplicity of cancers, the dataset lacks detailed oncology-related clinical information—such as cancer stage at diagnosis, treatment modalities, recurrence or progression status and treatment-related toxicities. These factors are strongly associated with long-term prognosis and may also influence metabolic status, thereby potentially modifying the observed association between BMR and mortality. Consequently, our estimates may be subject to unmeasured confounding and may not fully reflect heterogeneity across clinical trajectories. Future studies with linkage to cancer registries or electronic medical records, enabling adjustment for stage, treatment and recurrence, are warranted to validate and refine our findings. Third, the single-time-point estimation of BMR using predictive equations cannot capture its dynamic changes over time in cancer survivors. Although estimating BMR through predictive formulas was widely used and convenient, it may not accurately capture individual metabolic variations compared with direct or indirect calorie measurements [45]. Fourth, the limited number of cardiovascular death samples from individuals under the age of 60 restricted in-depth analysis of the younger subgroup, potentially affecting the reliability of the findings. Fifth, the generalizability of our findings is limited to the U.S. population, and their applicability to other countries or ethnic groups requires further investigation. Sixth, because BMR was estimated using a deterministic equation, its incremental prognostic value beyond its components or simpler measures is not established by this study. Direct assessments of body composition or cachexia were also lacking. Future studies using measured energy expenditure and detailed phenotyping are needed to address these questions. Seventh, cancer diagnoses were primarily based on self-reports, which are subject to misclassification due to recall bias. Additionally, the use of multiple imputation for missing data may introduce selection bias if the data are not missing at random, thereby limiting the generalizability of the findings.

## Conclusion

In conclusion, our study demonstrated that equation-estimated BMR is lower in cancer survivors and that its association with mortality is modified by age and gender, manifesting as a U-shaped relationship in older women, a linear inverse association in older men and a null association in younger survivors. These findings suggest that BMR may reflect underlying metabolic reserve and highlight the importance of considering age and sex when interpreting metabolic markers in survivorship. These findings are exploratory and should not be interpreted as direct clinical guidance.

## Supplementary Material

supplementary data.docx

## Data Availability

The data of this study are available from the corresponding author upon reasonable request. In addition, the original data are publicly accessible on the NHANES website.

## References

[1] Bizuayehu HM, Ahmed KY, Kibret GD, et al. Global disparities of cancer and its projected burden in 2050. JAMA Netw Open. 2024;7(11):e2443198. doi:10.1001/jamanetworkopen.2024.43198. 39499513 PMC11539015

[2] Siegel RL, Kratzer TB, Giaquinto AN, et al. Cancer statistics, 2025. CA Cancer J Clin. 2025;75(1):10–45. doi:10.3322/caac.21871. 39817679 PMC11745215

[3] Wagle NS, Nogueira L, Devasia TP, et al. Cancer treatment and survivorship statistics, 2025. CA Cancer J Clin. 2025;75(4):308–340. doi:10.3322/caac.70011. 40445120 PMC12223361

[4] Jensen RE, Potosky AL, Moinpour CM, et al. United States population-based estimates of patient-reported outcomes measurement information system symptom and functional status reference values for individuals with cancer. J Clin Oncol. 2017;35(17):1913–1920. doi:10.1200/JCO.2016.71.4410. 28426375 PMC5466008

[5] Sun KX, Liang X, Zhu Q, et al. Global patterns and trends in cancer-related premature death and their impact on life expectancy across 185 countries: a population-based analysis. Mil Med Res. 2025;12(1):56. doi:10.1186/s40779-025-00645-9. 40903768 PMC12406598

[6] Chen Y, Zhang J, Li Z, et al. Causal relationship between basal metabolic rate and kidney stone disease: from discovery in US NHANES to evidence in UK Biobank cohorts. Int J Surg. 2025;111(9):6063–6074. doi:10.1097/JS9.0000000000002658. 40497773 PMC12430904

[7] Kitazoe Y, Kishino H, Tanisawa K, et al. Renormalized basal metabolic rate describes the human aging process and longevity. Aging Cell. 2019;18(4):e12968. doi:10.1111/acel.12968. 31187606 PMC6612648

[8] Kliemann N, Murphy N, Viallon V, et al. Predicted basal metabolic rate and cancer risk in the European Prospective Investigation into Cancer and Nutrition. Int J Cancer. 2020;147(3):648–661. doi:10.1002/ijc.32753. 31652358

[9] Maciak S, Sawicka D, Kasacka I, et al. Basal metabolic rate shapes the development and progression of hepatocellular carcinoma. BMC Cancer. 2025;25(1):1102. doi:10.1186/s12885-025-14491-4. 40598035 PMC12210623

[10] Bartke A, Brannan S, Hascup E, et al. Energy metabolism and aging. World J Mens Health. 2021;39(2):222–232. doi:10.5534/wjmh.200112. 33151044 PMC7994661

[11] Ruggiero C, Ferrucci L. The endeavor of high maintenance homeostasis: resting metabolic rate and the legacy of longevity. J Gerontol A Biol Sci Med Sci. 2006;61(5):466–471. doi:10.1093/gerona/61.5.466. 16720742 PMC2645618

[12] Chen K, Zhang Y, Zhou S, et al. The association between the basal metabolic rate and cardiovascular disease: a two-sample Mendelian randomization study. Eur J Clin Invest. 2024;54(5):e14153. doi:10.1111/eci.14153. 38229569

[13] Maciak S, Sawicka D, Sadowska A, et al. Low basal metabolic rate as a risk factor for development of insulin resistance and type 2 diabetes. BMJ Open Diabetes Res Care. 2020;8(1):e001381. doi:10.1136/bmjdrc-2020-001381. PMC7373309 32690630

[14] Flores Ruano T, Hoogendijk EO, Romero Rizos L, et al. Resting metabolic rate in relation to incident disability and mobility decline among older adults: the modifying role of frailty. Aging Clin Exp Res. 2023;35(3):591–598. doi:10.1007/s40520-022-02340-4. 36626043

[15] Han F, Hu F, Wang T, et al. Association between basal metabolic rate and all-cause mortality in a prospective cohort of southern chinese adults. Front Physiol. 2021;12:790347. doi:10.3389/fphys.2021.790347. 35058799 PMC8763786

[16] Ruggiero C, Metter EJ, Melenovsky V, et al. High basal metabolic rate is a risk factor for mortality: the baltimore longitudinal study of aging. J Gerontol A Biol Sci Med Sci. 2008;63(7):698–706. doi:10.1093/gerona/63.7.698. 18693224 PMC4984846

[17] Jumpertz R, Hanson RL, Sievers ML, et al. Higher energy expenditure in humans predicts natural mortality. J Clin Endocrinol Metab. 2011;96(6):E972–976. doi:10.1210/jc.2010-2944. 21450984 PMC3100751

[18] Schrack JA, Knuth ND, Simonsick EM, et al. “IDEAL” aging is associated with lower resting metabolic rate: the baltimore longitudinal study of aging. J Am Geriatr Soc. 2014;62(4):667–672. doi:10.1111/jgs.12740. 24635835 PMC3989425

[19] Wu N, Zhu Y, Kadel D, et al. The prognostic influence of body mass index, resting energy expenditure and fasting blood glucose on postoperative patients with esophageal cancer. BMC Gastroenterol. 2016;16(1):142. doi:10.1186/s12876-016-0549-6. 28003023 PMC5175390

[20] Huang YS, Zeng XY, Chen WS, et al. Correlation between basal metabolic rate and clinical outcomes in gastric cancer patients: a retrospective study. J Invest Surg. 2024;37(1):2350358. doi:10.1080/08941939.2024.2350358. 38724045

[21] An S, Eo W, Kim SB, et al. Basal metabolic rate by FAO/WHO/UNU as a prognostic factor for survival in patients with gastric cancer: a cohort study. Medicine (Baltimore). 2024;103(47):e40665. doi:10.1097/MD.0000000000040665. 39809163 PMC11596422

[22] Nixon DW, Kutner M, Heymsfield S, et al. Resting energy expenditure in lung and colon cancer. Metabolism. 1988;37(11):1059–1064. doi:10.1016/0026-0495(88)90068-6. 3185289

[23] Vazeille C, Jouinot A, Durand JP, et al. Relation between hypermetabolism, cachexia, and survival in cancer patients: a prospective study in 390 cancer patients before initiation of anticancer therapy. Am J Clin Nutr. 2017;105(5):1139–1147. doi:10.3945/ajcn.116.140434. 28356274

[24] Langius JAE, Kruizenga HM, Uitdehaag BMJ, et al. Resting energy expenditure in head and neck cancer patients before and during radiotherapy. Clin Nutr. 2012;31(4):549–554. doi:10.1016/j.clnu.2011.12.009. 22265724

[25] Johnstone AM, Murison SD, Duncan JS, et al. Factors influencing variation in basal metabolic rate include fat-free mass, fat mass, age, and circulating thyroxine but not sex, circulating leptin, or triiodothyronine. Am J Clin Nutr. 2005;82(5):941–948. doi:10.1093/ajcn/82.5.941. 16280423

[26] Purcell SA, Marker RJ, Cornier MA, et al. Dietary intake and energy expenditure in breast cancer survivors: a review. Nutrients. 2021;13(10):3394. doi:10.3390/nu13103394. 34684403 PMC8540510

[27] Purcell SA, Wallengren O, Baracos VE, et al. Determinants of change in resting energy expenditure in patients with stage III/IV colorectal cancer. Clin Nutr. 2020;39(1):134–140. doi:10.1016/j.clnu.2018.12.038. 30975554

[28] Prado CMM, Lieffers JR, McCargar LJ, et al. Prevalence and clinical implications of sarcopenic obesity in patients with solid tumours of the respiratory and gastrointestinal tracts: a population-based study. Lancet Oncol. 2008;9(7):629–635. doi:10.1016/S1470-2045(08)70153-0. 18539529

[29] Martin L, Birdsell L, Macdonald N, et al. Cancer cachexia in the age of obesity: skeletal muscle depletion is a powerful prognostic factor, independent of body mass index. J Clin Oncol. 2013;31(12):1539–1547. doi:10.1200/JCO.2012.45.2722. 23530101

[30] Guida JL, Ahles TA, Belsky D, et al. Measuring aging and identifying aging phenotypes in cancer survivors. J Natl Cancer Inst. 2019;111(12):1245–1254. doi:10.1093/jnci/djz136. 31321426 PMC7962788

[31] Haupt S, Caramia F, Klein SL, et al. Sex disparities matter in cancer development and therapy. Nat Rev Cancer. 2021;21(6):393–407. doi:10.1038/s41568-021-00348-y. 33879867 PMC8284191

[32] Austad SN. Why women live longer than men: sex differences in longevity. Gend Med. 2006;3(2):79–92. doi:10.1016/s1550-8579(06)80198-1. 16860268

[33] Ko SH, Jung Y. Energy metabolism changes and dysregulated lipid metabolism in postmenopausal women. Nutrients. 2021;13(12):4556. doi:10.3390/nu13124556. 34960109 PMC8704126

[34] Kensler KH, Eliassen AH, Rosner BA, et al. Pre-diagnostic sex hormone levels and survival among breast cancer patients. Breast Cancer Res Treat. 2019;174(3):749–758. doi:10.1007/s10549-018-05121-8. 30604001 PMC6446911

[35] Janssen I, Heymsfield SB, Wang ZM, et al. Skeletal muscle mass and distribution in 468 men and women aged 18-88 yr. J Appl Physiol (1985). 2000;89(1):81–88. doi:10.1152/jappl.2000.89.1.81. 10904038

[36] Argilés JM, Campos N, Lopez-Pedrosa JM, et al. Skeletal muscle regulates metabolism via interorgan crosstalk: roles in health and disease. J Am Med Dir Assoc. 2016;17(9):789–796. doi:10.1016/j.jamda.2016.04.019. 27324808

[37] Seyfried TN, Shelton LM. Cancer as a metabolic disease. Nutr Metab (Lond). 2010;7(1):7. doi:10.1186/1743-7075-7-7. 20181022 PMC2845135

[38] Zhang Y, Yang JM. Altered energy metabolism in cancer: a unique opportunity for therapeutic intervention. Cancer Biol Ther. 2013;14(2):81–89. doi:10.4161/cbt.22958. 23192270 PMC3572003

[39] Ng JCM, Schooling CM. Sex-specific Mendelian randomization phenome-wide association study of basal metabolic rate. Sci Rep. 2025;15(1):14368. doi:10.1038/s41598-025-98017-9. 40274879 PMC12022104

[40] Georgopoulos NA, Saltamavros AD, Vervita V, et al. Basal metabolic rate is decreased in women with polycystic ovary syndrome and biochemical hyperandrogenemia and is associated with insulin resistance. Fertil Steril. 2009;92(1):250–255. doi:10.1016/j.fertnstert.2008.04.067. 18678372

[41] Sun R, Wang J, Li M, et al. Association of insulin resistance with cardiovascular disease and all-cause mortality in type 1 diabetes: systematic review and meta-analysis. Diabetes Care. 2024;47(12):2266–2274. doi:10.2337/dc24-0475. 39018337

[42] Penno G, Solini A, Orsi E, et al. Insulin resistance, diabetic kidney disease, and all-cause mortality in individuals with type 2 diabetes: a prospective cohort study. BMC Med. 2021;19(1):66. doi:10.1186/s12916-021-01936-3. 33715620 PMC7962330

[43] Fearon K, Arends J, Baracos V. Understanding the mechanisms and treatment options in cancer cachexia. Nat Rev Clin Oncol. 2013;10(2):90–99. doi:10.1038/nrclinonc.2012.209. 23207794

[44] Argilés JM, Busquets S, Stemmler B, et al. Cancer cachexia: understanding the molecular basis. Nat Rev Cancer. 2014;14(11):754–762. doi:10.1038/nrc3829. 25291291

[45] Lam YY, Ravussin E. Indirect calorimetry: an indispensable tool to understand and predict obesity. Eur J Clin Nutr. 2017;71(3):318–322. doi:10.1038/ejcn.2016.220. 27848941

