# Supplementary material for: Association of basal metabolic rate with mortality among cancer survivors: a cohort study emphasizing heterogeneity by gender and age
Source: Ann Med. 2026 Aug 5;58(1):2707731. doi: 10.1080/07853890.2026.2707731 (PMC13446061; doi:10.1080/07853890.2026.2707731)
Supplement: Supplementary file 1 [file IANN_A_2707731_SM3887.docx]

Supplementary Table 1 Distribution of variables with missing data

| **Variables** | **Number of missing** | **Missing proportion** |
| --- | --- | --- |
| Drinking status | 4580 | 9.99% |
| PIR | 3880 | 8.46% |
| WC | 2016 | 4.40% |
| Education | 55 | 0.12% |
| first diagnosis year^a^ | 39 | 1.14% |
| Smoking status | 31 | 0.07% |
| Marital status | 28 | 0.06% |
| Hypertension | 15 | 0.03% |
| CVD | 4 | 0.01% |
| Activity | 3 | 0.01% |
| Diabetes | 0 | 0 |
| Race | 0 | 0 |
| BMI | 0 | 0 |
| Cancer number | 0 | 0 |

Supplementary Table 2 Multivariable-adjusted linear regression model of BMR among US cancer survivorsa

| **Variables** | **β** | **95% CI** | **P-value** |
| --- | --- | --- | --- |
| Gender |  |  |  |
| Male | Reference (0) |  |  |
| Female | -6.26 | -6.46,-6.05 | <0.001 |
| Age |  |  |  |
| <60years | Reference (0) |  |  |
| ≥60 years | -0.16 | -0.17,-0.16 | <0.001 |
| Race |  |  |  |
| Mexican American | Reference (0) |  |  |
| Other Hispanic | -0.17 | -0.62, 0.29 | 0.478 |
| Non-Hispanic White | 0.7 | 0.34, 1.05 | <0.001 |
| Non-Hispanic Black | 1.6 | 1.16, 2.04 | <0.001 |
| Other Race | 0.56 | -0.12, 1.25 | 0.104 |
| Educational attainment |  |  |  |
| <High school | Reference (0) |  |  |
| High school | 0.46 | 0.22, 0.70 | <0.001 |
| >High school | 0.74 | 0.49, 0.99 | <0.001 |
| Marital status |  |  |  |
| Married/living with partner | Reference (0) |  |  |
| Widowed/divorced/separated | -0.61 | -0.82, -0.39 | <0.001 |
| Never married | 0.25 | -0.29, 0.78 | 0.362 |
| PIR |  |  |  |
| Low(<1) | Reference (0) |  |  |
| Medium(1-3) | -0.49 | -0.84, -0.13 | 0.008 |
| High(>3) | 0.08 | -0.24, 0.40 | 0.602 |
| Drinking status |  |  |  |
| No | Reference (0) |  |  |
| Yes | 0.36 | 0.10, 0.61 | 0.006 |
| Smoking status |  |  |  |
| Never smoker | Reference (0) |  |  |
| Former smoker | -0.08 | -0.30, 0.15 | 0.508 |
| Current smoker | 0.41 | 0.10, 0.72 | 0.01 |
| Hypertension |  |  |  |
| No | Reference (0) |  |  |
| Yes | -0.60 | -0.83,-0.37 | <0.001 |
| Diabetes |  |  |  |
| No | Reference (0) |  |  |
| Yes | -0.05 | -0.33,0.23 | 0.709 |
| CVD |  |  |  |
| No | Reference (0) |  |  |
| Yes | -0.62 | -0.85,-0.39 | <0.001 |
| Physical activity |  |  |  |
| Inactive | Reference (0) |  |  |
| Active | 0.36 | 0.14,0.58 | 0.001 |
| BMI |  |  |  |
| <25 | Reference (0) |  |  |
| 25-30 | 0.87 | 0.61, 1.13 | <0.001 |
| ≥30 | 2.58 | 2.15, 3.00 | <0.001 |

^a^: Weighted to be nationally representative. Adjusted for gender, age, race, marital status, educational attainment, and poverty income ratio, waist circumference, body mass index, physical activity, drinking status, and smoking status, hypertension, diabetes, cardiovascular disease. β-coefficients were estimated for a per 50-unit increase in BMR. BMR=basal metabolic rate; PIR, poverty income ratio; BMI, body mass index; CVD, cardiovascular disease.

Supplementary Table 3 Subgroup analysis of the association between BMR and all-cause and cause-specific mortality among cancer survivors, stratified by gender and age(weighted)

| **Mortality outcome** | **Male and < 60 years at interview** | | **Male and ≥ 60 years at interview** | | **Female and < 60 years at interview** | | **Female and ≥ 60 years at interview** | |
| --- | --- | --- | --- | --- | --- | --- | --- | --- |
|  | **Death(n/N)** | **HR(95% CI)** | **Death(n/N)** | **HR(95% CI)** | **Death(n/N)** | **HR(95% CI)** | **Death(n/N)** | **HR(95% CI)** |
| **All cause** | | | | | | | | |
| Per 50 kcal/d increase | 52/250 | 0.99(0.87,1.14) | 593/1271 | 0.95(0.91,1.00) | 54/763 | 0.96(0.85,1.08) | 446/1177 | 0.85(0.81,0.89)*** |
| Q1 |  | Reference |  | Reference |  | Reference |  | Reference |
| Q2 |  | 0.82(0.33,2.03) |  | 0.89(0.65,1.21) |  | 1.41( 0.56, 3.57) |  | 0.53( 0.40, 0.71)*** |
| Q3 |  | 0.62(0.18,2.15) |  | 0.62(0.43,0.89)** |  | 2.67( 0.69, 10.34) |  | 0.29( 0.20, 0.42)*** |
| Q4 |  | 0.89(0.16,4.92) |  | 0.71(0.45，1.11) |  | 2.66(0.62, 11.39) |  | 0.20(0.12, 0.31)*** |
| p for trend |  | 0.627 |  | 0.042 |  | 0.191 |  | <0.001 |
| **Cardiac disease** | | | | | | | | |
| Per 50 kcal/d increase | 3/250 | NA | 132/1271 | 0.86(0.78,0.96)** | 7/763 | NA | 88/1177 | 0.82(0.74,0.91)*** |
| Q1 |  | NA |  | Reference |  | NA |  | Reference |
| Q2 |  | NA |  | 0.82(0.47,1.41) |  | NA |  | 0.40(0.21,0.76)** |
| Q3 |  | NA |  | 0.30(0.15,0.61)*** |  | NA |  | 0.18(0.07,0.47)*** |
| Q4 |  | NA |  | 0.19(0.07,0.50)*** |  | NA |  | 0.07(0.03,0.17)*** |
| p for trend |  | NA |  | <0.001 |  | NA |  | <0.001 |
| **Cancer** | | | | | | | | |
| Per 50 kcal/d increase | 28/250 | 0.98(0.77,1.25) | 219/1271 | 1.02(0.94,1.10) | 30/763 | 0.91(0.74,1.11) | 130/1177 | 0.95(0.87,1.05) |
| Q1 |  | Reference |  | Reference |  | Reference |  | Reference |
| Q2 |  | 1.65(0.37,8.42) |  | 0.83(0.50,1.37) |  | 2.86(0.64,12.72) |  | 0.71(0.41,1.21) |
| Q3 |  | 0.48(0.09,2.76) |  | 0.76(0.42,1.37) |  | 4.31(0.39,47.69) |  | 0.53(0.25,1.14) |
| Q4 |  | 0.68(0.06,10.17) |  | 1.14(0.58,2.24) |  | 2.41(0.19,30.34) |  | 0.47(0.19,1.14) |
| p for trend |  | 0.699 |  | 0.924 |  | 0.568 |  | 0.084 |
| **All other causes** | | | | | | | | |
| Per 50 kcal/d increase | 21/250 | 0.94(0.74,1.18) | 242/1271 | 0.92(0.86,0.99)* | 17/763 | 1.17(0.94，1.45） | 228/1177 | 0.84(0.79,0.91)*** |
| Q1 |  | Reference |  | Reference |  | Reference |  | Reference |
| Q2 |  | 0.26(0.04,1.74) |  | 0.97(0.60,1.58) |  | 0.84(0.13, 5.31) |  | 0.54(0.36, 0.80)** |
| Q3 |  | 1.06(0.15,7.60) |  | 0.75(0.43,1.30) |  | 4.62(0.66, 32.25) |  | 0.25(0.15, 0.41)*** |
| Q4 |  | 0.16(0.01,4.26) |  | 0.81(0.41,1.61) |  | 11.62(1.63, 82.87)* |  | 0.18(0.09, 0.36)*** |
| p for trend |  | 0.776 |  | 0.407 |  | 0.044 |  | <0.001 |

weighted:Adjusted for race, marital status, educational attainment, and poverty income ratio, waist circumference, body mass index, physical activity, drinking status, and smoking status, hypertension, diabetes, cardiovascular disease, years since first diagnosis of cancer, age of first cancer diagnosis, and number of cancer types.

BMR=basal metabolic rate; HR=hazard ratio; CI=confidence interval; NA=not applicable

Significance levels: ***P < 0.001, **P < 0.01, *P < 0.05.

Supplementary Table 4 Threshold effect analysis of BMR on all-cause and cardiac mortality in cancer survivors (Female and ≥ 60 year)

| **Mortality outcome** | **Death (n/N)** | **Adjusted HR (95% CI)** | **p-Value** |
| --- | --- | --- | --- |
| **All-cause mortality** | | | |
| Inflection point, kcal/d | 1546.8 | | |
| BMR <1546.8 | 422/1086 | 0.84 (0.81,0.88) | <0.001 |
| BMR ≥1546.8 | 24/91 | 1.49 (1.26,1.77) | <0.001 |
| P for Log-likelihood ratio | 0.001 | | |
| **Cardiac mortality** | | | |
| Inflection point, kcal/d | 1552.1 | | |
| BMR <1552.1 | 83/1089 | 0.77 (0.69,0.85) | < 0.001 |
| BMR ≥1552.1 | 5/88 | NA | NA |
| P for Log-likelihood ratio | 0.153 | | |

Adjusted for race, marital status, educational attainment, and poverty income ratio, waist circumference, body mass index, physical activity, drinking status, and smoking status, hypertension, diabetes, and cardiovascular disease, years since first diagnosis of cancer, age of first cancer diagnosis, and number of cancer types.

BMR=basal metabolic rate; HR=hazard ratio; CI = confidence interval.

Supplementary Figure 1 Flow chart of participant selection: Inclusion and exclusion criteria for the NHANES 2001-2018 analysis

Supplementary Figure 2 Forest plot of association between basal metabolic rate(BMR) and all cause mortality. Subgroup analysis was stratified by different cancer types, years since first diagnosis of cancer, and age at first cancer diagnosis

Supplementary Figure 3 Forest plot of association between basal metabolic rate(BMR) and all cause mortality. Subgroup analysis was stratified by gender, age, race, educational attainment, marital status, physical activity, body mass index (BMI), hypertension, and diabetes, smoking status, alcohol consumption and cardiovascular disease(CVD)
